# Development and evaluation of an internet-based cognitive behavioral therapy intervention for anxiety and depression in adults with cystic fibrosis (eHealth CF-CBT): An international collaboration

**DOI:** 10.1016/j.invent.2021.100372

**Published:** 2021-02-24

**Authors:** Marieke Verkleij, Anna M. Georgiopoulos, Deborah Friedman

**Affiliations:** a Department of Pediatric Psychology, Amsterdam UMC, Vrije Universiteit Amsterdam, Amsterdam, the Netherlands; b Department of Psychiatry, Massachusetts General Hospital, Boston, MA, USA

**Keywords:** Depression, Anxiety, Cystic fibrosis, Psychotherapy, Cognitive-behavioral therapy, Telehealth

## Abstract

**Introduction:**

Individuals with cystic fibrosis (CF) are at increased risk for anxiety and depression, with negative consequences for adherence, health, and quality of life. New approaches to prevent and treat anxiety and depression that are tailored to the concerns of this population are needed. A CF-specific internet-based cognitive-behavioral therapy (CBT) intervention was developed to increase access to evidence-based mental health care and decrease cost and burden of care for people with CF.

**Objective:**

To evaluate the usability and acceptability of “eHealth CF-CBT,” an internet-based program integrating therapist-guided online self-management modules with in-person or virtual sessions.

**Methods:**

Dutch adults with CF (*N* = 16) and CF health care providers (N = 16) systematically tested all sessions of the eHealth CF-CBT program, provided qualitative feedback, and completed measures including eHealth Impact Questionnaire (eHIQ), and System Usability Scale (SUS).

**Results:**

Patient and provider ratings of their overall impression of the eHealth CF-CBT program were high, with scores (mean; SD) of 8.3; 0.6 and 8.2; 0.8 respectively on a 10-point scale. Mean ratings of usability by patients (77.0/100) and providers (73.4/100) surpassed the SUS cut point for good favorability. Ratings (pooled mean; SD) on the assessed eHIQ domains Motivation and Confidence to Act (71.3; 10.0), Information and Presentation (78.9; 9.6), and Identification (62.0; 15.1) were positive, as were assessments of specific elements of session content and format.

**Conclusions:**

eHealth CF-CBT is the first therapist-guided internet-delivered CBT-based intervention for adults with CF. Initial evaluation with key stakeholders demonstrated high levels of acceptability and usability and provided input that was integrated to strengthen the program. Effectiveness testing in the Netherlands will be the next step, as well as future international adaptation and dissemination.

## Highlights

• There is an urgent need for psychological interventions that reduce barriers to care for people with cystic fibrosis (CF).
• The eHealth CF-CBT program is the first therapist-guided internet-delivered CBT-based intervention for adults with CF.
• Initial evaluation of eHealth CF-CBT with patients and providers demonstrated high levels of acceptability and usability.
• Qualitative input was integrated in preparation for effectiveness testing and international adaptation and dissemination.

## 1 Introduction

Cystic Fibrosis (CF) is a progressive, multisystem genetic disease affecting more than 70,000 individuals worldwide, often marked by difficulty breathing due to recurrent pulmonary infections and damage (Rayment, 2020). Despite treatment advances that have increased average life expectancy, living with CF entails a high burden of distressing symptoms and daily treatments (Friedman et al., 2018a; Sawicki et al., 2008). TIDES-CF, an epidemiological study of over 6000 individuals with CF, found elevated rates of depression and anxiety 2–3 times more common in individuals with CF than in the general population (Quittner et al., 2014). Moreover, untreated depression and anxiety in people with CF were found to have negative consequences for health-related quality of life (HrQoL), adherence, medical outcomes and healthcare costs (Quittner et al., 2014; Smith et al., 2010; Verkleij et al., 2018). Consequently, international mental health guidelines developed by the CF Foundation (CFF) and European CF Society (ECFS) recommend routine screening, treatment, and preventative efforts for depression and anxiety as a component of standard CF care (Abbott et al., 2019; Quittner et al., 2016).

However, there are unique barriers related to health care burden and infection risk that may impede access to mental health care in this population. Individuals with CF face a high burden of medical treatment including a self-care regimen that averages between an hour to 2 h a day and frequent medical appointments. Hospitalizations lasting two weeks or more to treat exacerbations requiring IV antibiotics are common, and may be disruptive to regular outpatient mental health care (Mueller et al., 2020). Even before the pandemic, individuals with CF have had to practice strict infection control due to vulnerability to infections leading to illness and reduction in lung functioning over time (Saiman et al., 2014). Furthermore, infection control guidelines for individuals with CF recommend that individuals with CF avoid being in the same room and maintain 6 ft of distance from others with CF due to high risk associated with cross-infection (Saiman et al., 2014). In addition, community-based mental health care providers may have little to no knowledge of CF, placing the burden on individuals with CF to educate their providers about the disease (Quittner et al., 2016). For these reasons, there is increased burden and risk associated with mental health care appointments, and in-person group care models in CF are contraindicated. Individuals with CF may prefer and engage more fully in mental health care that is integrated into their routine CF care, delivered by mental health providers embedded within their care team, and may find integrated care that is available via telehealth or internet easier to access (Abbott et al., 2019; Quittner et al., 2020). Internet-delivered psychosocial interventions might be especially relevant and suitable for patients with CF to decrease healthcare burden and address the many barriers that exist to accessing mental health care (Quittner et al., 2016).

Cognitive-behavioral therapy (CBT), an evidence-based structured mental health intervention focused on teaching adaptive coping skills, was recommended in the CFF/ECFS guidelines for those screening with elevated depression or anxiety symptoms (Quittner et al., 2016). However, while there are effective chronic illness condition-specific CBT protocols for a number of medical conditions, including diabetes, COPD, HIV/AIDS, epilepsy, and cancer (Antoni et al., 2006, Antoni et al., 2009; Cully et al., 2010; Laperriere et al., 2005; Thompson et al., 2010; Safren et al., 2009; Soo and Lam, 2009), until recently, there were no interventions tailored to address the unique needs of individuals with CF.

To respond to an urgent need for evidence-based psychological interventions for people with CF (Ammerlaan et al., 2017; Verkleij et al., 2017), CF-CBT, an 8-session modular manualized intervention that could be integrated into routine CF care was developed in the US with input from individuals with CF and CF healthcare providers (Friedman and Georgiopoulos, 2016). As an international collaboration across the US and the Netherlands, the CF-CBT program was then adapted into an internet-based program, eHealth CF-CBT. The eHealth CF-CBT program, available in both English and Dutch, is a blended care program integrating therapist-guided online self-management modules with in-person or virtual sessions. Patients can access eHealth CF-CBT from home while continuing to benefit from the expert guidance and feedback of a mental health provider, reducing barriers including transportation and other costs of care. The eHealth CBT program will also be adaptable for dissemination to countries with a wide range of resource availability and healthcare systems. The aim of this study was to evaluate the usability and acceptability of “eHealth CF-CBT” with key stakeholders.

## 2 Methods

### 2.1 Program development and evaluation protocol

#### 2.1.1 CF-CBT program (manualized version)

As noted, to address the need for accessible evidence-based psychological interventions to prevent and treat depression and anxiety for people with CF, a CF-specific CBT-based intervention manual, workbook, and training program were developed and designed to be integrated into existing CF care systems, “CF-CBT: A cognitive-behavioral skills-based program to promote emotional well-being for adults with CF” (Friedman, 2019; Friedman et al., 2019). The program was developed in partnership, with input and feedback from adults with CF and CF healthcare providers, and specifically tailored to address the emotional challenges of living with CF. Direct quotes from patients with CF who participated in the development project were included in the program materials. The intervention consists of eight 45-minute sessions that can be flexibly delivered in the outpatient CF clinic, on the inpatient unit, or by telephone to increase accessibility. The original CF-CBT was developed as a preventive intervention, targeted for CF adults who screen in the mild range for depression and/or anxiety according to the CFF/ECFS guidelines (Abbott et al., 2019; Quittner et al., 2016), that could be provided by multidisciplinary CF health-care providers from a variety of disciplines (nurses, social workers, psychologists, psychiatrists, or pulmonologists), including mental health and non-mental health clinicians, with specialized training in the provision of the intervention.

The eight sessions of CF-CBT cover skills including relaxation and mindfulness, behavioral activation, anxiety management skills, cognitive restructuring, and health-related goal setting and problem-solving. Each session includes teaching on the session topic, a skill-building exercise, and an experiential homework assignment to practice applying each skill in-between sessions.

CF-CBT was piloted at 3 US CF centers and was well received by individuals with CF and interventionists (Friedman et al., 2018b). Results of the pilot study found CF-CBT to be feasible, acceptable to adults with CF and to have promising preliminary effectiveness outcomes, with decreases in depression, anxiety, and perceived stress, and improvements in quality of life and coping skills (Friedman et al., 2019). A 3-year multicenter waitlist-controlled randomized controlled trial, and the development and pilot of an adaptation developmentally appropriate for adolescents, are currently ongoing (Cystic Fibrosis Foundation Therapeutics, Inc.; Friedman and Georgiopoulos, PI's).

### 2.2 Development of eHealth CF-CBT in English and Dutch

The aim of this project was to develop a modular, CBT-informed, CF team-guided, internet delivered intervention for prevention and treatment of depression and anxiety, customized to the concerns of adults with CF and models of healthcare delivery in the US and the Netherlands, eHealth CF-CBT. The CF-CBT program was built for integration into the model of CFF accredited care teams in the US and required linguistic, cultural, and structural modification prior to international dissemination. A therapist-guided blended care model that involves online self-management modules along with therapist-led modules reduces the need for in-person appointments and thereby, may increase accessibility for individuals with CF and feasibility for CF care teams. The CF-CBT intervention manual was thus adapted to eHealth CF-CBT, a therapist-guided internet-delivered program for adults with CF, in English and Dutch, according to the following steps.

First, the CF-CBT manualized program was translated into Dutch and culturally adapted for use in the Netherlands. Cultural adaptations included the following:

• Text referencing the healthcare system, insurance and healthcare costs was altered to reflect healthcare system differences between the US and the Netherlands, where healthcare insurance is obligatory for every inhabitant and most medical costs are covered.
• Content related to delivery of mental health care was made appropriate to the local context. In the Netherlands, a psychologist and/or social worker are part of the multidisciplinary CF care team in every Dutch CF center. Psychological care available within the CF care team as well as referral pathways for psychological care outside the clinic may differ between Dutch and US CF centers.
• Different resources, such as relaxation apps and mental health-related websites, were referenced. In particular, there are more options for eHealth interventions available in the Netherlands. Resources provided in English, such as relaxation exercises, were re-recorded in Dutch, and identified websites and resources for follow-up care were modified to fit the appropriate context.
• The nuances of language were also adapted in some instances to a more down-to-earth, less enthusiastic tone to reflect Dutch cultural norms (e.g. when acknowledging or congratulating participants for session completion).

Next, the authors created CF team guided, internet-delivered adaptations of the manualized CF-CBT program in English (eHealth CF-CBT). The 8 modules of the online CF-CBT program are delivered through blended care: online content completed during in-person or telehealth sessions with the therapist in combination with self-guided online modules. The initial intake, fourth and last sessions are designed to be completed in real time with the therapist. The remaining 5 sessions are self-guided, with online content completed by the patient on their own, and e-mail feedback on completed skills-based homework assignments subsequently provided by the therapist.

To build the eHealth CF-CBT program, the e-health platform Minddistrict was used (https://www.minddistrict.com/). It is important for any online platform to be easily and inexpensively embedded in the CF care system (Abbott et al., 2019; Quittner et al., 2020), and the Minddistrict platform is already established in the Netherlands. The Minddistrict eHealth platform allows for incorporation of interactive text, audio and videos, integrating questionnaires, daily diaries, and clinician monitoring and feedback. Patients receive online access for at least half a year after finishing the intervention and can view and print the work they completed within the program. Clinician providers have access to an administrator page allowing them to view patients' progress and give feedback.

The following adaptations were made from the CF-CBT manual to the eHealth program:

• The visual aesthetic of the program became a much more salient feature. Photos were added throughout to illustrate the text and keep participants engaged in self-guided modules. A variety of fonts and colors were used to highlight text.
• A video using animation to introduce the program to participants was developed and included in an introduction module.
• The self-guided internet format required streamlining and organizing text (e.g. with bullet points). Participants are given the option to make hidden text appear to read further or receive more detailed explanation on some topics.
• Interactive text features were included such that participants receive immediate feedback based on their responses in some areas.
• “Diaries” were added that provide smart phone reminders to complete the homework exercise and allow the participants to complete the exercise in real-time on their device.

After refinement of eHealth CF-CBT in English, the Dutch-language adaptation was built into Minddistrict. The English and Dutch eHealth CF-CBT programs were further refined based on feedback from adults with CF and CF healthcare providers obtained via the study of usability and acceptability presented here. In anticipation of a pilot study at Amsterdam UMC funded by the Dutch CF Foundation to further examine feasibility, acceptability, and preliminary effectiveness outcomes, the program was iteratively modified to further improve relevance to the model of healthcare delivery in the Netherlands.

### 2.3 Usability and acceptability feedback on eHealth CF-CBT

Adults with CF and CF healthcare providers from 4 academic hospitals in the Netherlands were invited to participate in this evaluation study via an announcement on the Facebook page of the Dutch CF Foundation and via their networks. Participants provided written informed consent, received the link to the online program, log-in codes and a link to the evaluation survey regarding eHealth CF-CBT, and were instructed to complete the program in a self-guided fashion and subsequently, to complete the evaluation measures and feedback questionnaire. Patients were reimbursed € 50 for participation, while healthcare providers donated their time to provide feedback data.

### 2.4 Measures

#### 2.4.1 eHealth Impact Questionnaire (eHIQ)

The e-HIQ was designed to assess the effectiveness of web-based programs containing health information (Kelly et al., 2013). The eHIQ is a self-completed questionnaire consisting of two independently administered and scored scales (eHIQ-Part 1 and eHIQ-Part 2). Each item has a five-point response option ranging from “strongly disagree” to “strongly agree”. eHIQ-Part 1 (11 Items) assesses general attitudes towards using the internet to access health information. eHIQ-Part 2 (26 items) measures a participant's view on the specific health-related website he or she has just looked at (eHealth CF-CBT). For this study we only administered eHIQ-Part 2 which is comprised of 3 domains:

1) **Motivation and confidence to act** (10 items), e.g. “The website encourages me to take actions that could be beneficial to my health” and “The website gives me confidence that I am able to manage my health”;
2) **Information and presentation** (13 items), e.g. “The website includes useful tips on how to make life better” and “I can easily understand the information on the website”;
3) **Identification** (3 items), e.g. “I can identify with other people using the website” and “I feel I have a lot in common with other people using the website”.

Each scale was transformed to a 0–100 metric, where 0 = low perceived value of the internet (or website) for health, and 100 = high perceived benefit of using the internet (or website) in relation to health (Kelly et al., 2015). All domains of this measure have demonstrated good internal consistency, with Cronbach's alphas ≥ 0.77, and good test-retest reliability after a two-week interval, with intra-class correlation coefficients ranging from 0.76 to 0.91 for all domains (Kelly et al., 2015). Specifically in the three domains of Part 2, Cronbach's alphas were 0.92, 0.89 and 0.90 respectively (Kelly et al., 2015). For this study, we used the Dutch translation of the eHIQ (Neijenhuijs et al., 2019). The eHIQ is a continuous scale for which the authors determined that a score of 65 or greater is considered positive (Neijenhuijs et al., 2019; Talboom-Kamp et al., 2020).

#### 2.4.2 System Usability Scale (SUS)

The SUS provides a brief, reliable tool for assessing usability. It consists of a 10-item scale, e.g. “I felt very confident using eHealth CF-CBT” and “I thought eHealth CF-CBT was easy to use,” with a Likert Scale with five response options from “strongly disagree” to “strongly agree”. Originally created by Brooke (Brooke, 1996, Brooke, 2013), it is designed for evaluation of a wide variety of products and services, including hardware, software, mobile devices, websites and applications. A SUS score between 68.0 and 80.3 is considered good, above 80.3 excellent (Bangor et al., 2009).

#### 2.4.3 eHealth CF-CBT Feedback Questionnaire

This questionnaire, designed for this study, invited participants to provide their overall impression of the program as an open response question and separate rating using a 10-point scale (0 is “very bad,” 10 is “excellent”). In addition, participants provided detailed session-by-session feedback on the presentation of information and functionality of the program, rated on a Likert scale from 1 to 5 with response options rating from “strongly disagree” to “strongly agree”. Open-ended feedback on acceptability and suggested changes for each session was also invited.

## 3 Results

### 3.1 Participants

32 participants (16 adults with CF and 16 healthcare providers) provided feedback on the eHealth CF-CBT program. See Table 1A, Table 1B for descriptive demographic information regarding study participants. Mean age of patient participants was 35.4 (*SD* = 8.8, range 24–55 yrs.) and they were primarily female (63%). Number of CF-related hospitalizations in the past year ranged from 0 to 5; only one patient had >1 hospitalization and 4 patients have had a lung transplant. Mean BMI was 20.93 (*SD* = 2.0) ranging from 16.14–23.53 and mean FEV_1_ percent predicted was 72.0% (*SD* = 25.0) ranging from 28 to 98%, indicating a diversity of nutritional status and lung disease severity. Fifty percent of patients indicated a history of diagnosed anxiety disorder or depression, and 81% stated they had received psychological treatment in the past.

**Table 1A:** Patient participant demographics.

Patients *N* = 16	*Mean* (*SD*), range	
Age in years	35.4 (8.8), 24–55	
Body mass index (BMI)	20.9 (2.0), 16.1–23.5	
Gender	10 Female	
	6 Male	
Highest level of education	N (%)	
Low/middle/high^a^	2/4/10	(12.5/25/62.5)
Main employment status	N (%)	
Wage employed	4 (25)	
Self-employed	1 (6)	
Currently not working	5 (31)	
Volunteer work	4 (25)	
Work at home, e.g. household care	2 (13)	
Average working hours per week	N (%)	
0	7 (44)	
1–12	4 (25)	
13–24	3 (19)	
≥25	2 (12)	
Relational status	N (%)	
Single	6 (38)	
Living together/married	9 (56)	
Widowed	1 (6)	
Age at diagnosis (years)	N (%)	
0–6	13 (81)	
7–12	1 (6)	
13–30	0 (0)	
30–35	2 (13)	
Lung transplant recipient	N (%)	
	4 (25)	
Most recent forced expiratory volume percent predicted (FEV_1_%pred)	*Mean* (*SD*), range	
	72.0% (25.0), 28%–108%	
Number of CF-related hospitalizations in past year	N (%)	
0	10 (63)	
1	5 (31)	
≥2	1 (6)	
History of diagnosed anxiety disorder or depression	N (%)	
	8 (50)	
Previous psychological treatment (counseling, coaching, psychotherapy)	N (%)	
	13 (81)	

a Education level, “Low”: Primary school or lower vocational secondary education, “Middle”: intermediate general secondary education or intermediate vocational education, and “High”: higher general secondary education, higher vocational education, or university education.

**Table 1B:** Healthcare provider participant demographics.

Healthcare providers, N = 16	*Mean* (*SD*), range
Age in years	40.1 (13.3), 25–62
Gender	15 Female
	1 Male
Highest level of education	N
Low/middle/high^a^	0/0/16
Occupation	N (%)
Psychologist	7 (44)
Social worker	2 (13)
Physician/pulmonologist	1 (6)
Nurse	2 (13)
Researcher	2 (13)
Other	2 (13)

a Education level, “Low”: Primary school or lower vocational secondary education, “Middle”: intermediate general secondary education or intermediate vocational education, and “High”: higher general secondary education, higher vocational education, or university education.

Healthcare provider participants ranged in age from 27 to 62 yrs. (*Mean* = 40.1, *SD* = 13.3), and were also primarily female (94%). Fifty-seven percent of participating healthcare providers were mental health clinicians (psychologists or social workers) working as part of CF multidisciplinary teams, while other participating providers were CF physicians (6%), nurses (13%), pulmonology researchers (13%) and others (13%; e.g. a coordinator of the Dutch CF Foundation).

### 3.2 Acceptability and usability survey results

Results of the eHIQ indicated positive ratings (cut-off score of ≥65; Talboom-Kamp et al., 2020) on the domains “Motivation and confidence to act” and “Information and presentation” with means ranging from 70.5 to 82.1 (patients' scores) and 72.2 to 75.6 (healthcare providers). The domain “Identification” was rated 62.0. The mean SUS ratings for usability of 77.0 (patients, ranging from 27.5 to 95.0) and 73.4 (healthcare providers, ranging from 50.0 to 92.5) were above average, and reached a favorable cut-off score (Bangor et al., 2009).

Patient and healthcare provider participant ratings of their overall impression of the eHealth CF-CBT program were high, with mean scores of 8.3 and 8.2 respectively on a 10-point scale, ranging from 6 to 9. Overall, patients rated the eHealth CF-CBT program slightly more positively on all domains compared with the healthcare providers (Table 2). Open-ended feedback regarding the program as a whole, obtained from participants using the eHealth CF-CBT Feedback Questionnaire designed for this study, was largely positive; for representative examples, see Table 3.

**Table 2:** Acceptability and usability ratings by participant type.

	Patient (N = 16)	Healthcare provider (N = 16)	Total (*N* = 32)
	*Mean* (*SD*)	*Mean* (*SD*)	*Mean* (*SD*)
eHealth Impact Questionnaire (eHIQ)			
Motivation and confidence to act	70.5 (10.3)	72.2 (9.9)	71.3 (10.0)
Information and presentation	82.1 (7.9)	75.6 (10.2)	78.9 (9.6)
Identification	62.0 (19.5)	62.0 (9.6)	62. 0 (15.1)
System Usability Scale (SUS)	77.0 (16.3)	73.4 (11.5)	75.2 (14.0)
Overall impression of the eHealth CF-CBT program	8.3 (0.6)	8.2 (0.8)	8.2 (0.7)

Note: eHealth Impact Questionnaire ranges from 0 to 100 where higher scores indicate a higher level of acceptability; System Usability Scale ranges from 0 to 100 where higher scores indicate greater usability ratings; Overall impression scores ranges from 0 to 10 where higher scores indicate a better overall impression.

**Table 3:** Qualitative evaluation of the eHealth CF-CBT program: representative quotes.

General content • *‘User-friendly website. Good that it focuses on positive changes. Content is nice, with good exercises.’* • *‘A very good and impressive program which will become an important part of the treatment of CF.’* • *‘A nice program that connects and brings CF into therapy.’* • *‘A very nice program and I would like to get working on it myself. It is not that extensive, but there are different steps that can help you get further.’* • *‘I thought it was a refreshing and inspiring program and I hope that eventually it will be used broadly. It is not patronizing which is nice! It offers another perspective on my health and how to deal with this. Not from the perspective of physical, but from the perspective of mental health. Even in this testing phase, I will take something with me. Thanks for that.’* • *‘It is clear, it is positive….This could definitely help people with CF who experience anxiety or depression-related complaints.’* • *‘I personally do not have depression/anxiety…, but it was very meaningful to read about this.’* • *‘Very complete and accessible. Functions work well. Good that you have the option of reading about subjects that you want to know more about. Good that there are reminder systems for the exercises. Widely usable! I would like to work with it too!’*
General suggestions for improvement • *‘Use less jargon. Watch out for too much psychologist lingo.’* • *‘Use more bullet points and pictures.’* • *‘Sometimes there's a lot of text, and I wonder how carefully this will be read. Making the text shorter might be better.’* • *‘A lot of women in the pictures, add more pictures of men.’*
Session specific suggestions for improvement *Introduction* • *‘Make more clear what the goals and outcomes are (or could be) of following the program and how the different sessions can add to this.’* • *‘I felt that having a question about the situation at home was missing, which might be good to understand.’* *Session 1* • *‘Perhaps you can add that you don't have to fill in the exercise now, but [can do it] during the week when a stressful situation occurs.’* • *`I don't find the photo of a playing child on the beach appropriate for a program meant for adults with CF (some of them struggle with wishing to have a child).’* *Session 2* • *‘Note that you are able to learn how to relax, and this takes time.’* • *‘Maybe indicate that doing the exercises at a set time of the day, for example when you get back home from work or right before going to bed, will help you keep doing the exercises.’* *Session 3* • *‘I understand the idea of an activity plan, but if I don't feel well, I am unable to do anything, which in my case would just make me feel worse.’* • *‘Can you see the chosen activity on your phone? Otherwise people might forget to log in to the site. Perhaps you can add as a tip to write this in your planner.’* *Session 4 & 5* • *‘Nice exercise about recognizing thinking mistakes. Perhaps with the exercises you can have a fold-out text to look back to what constitutes the different thinking mistakes.’* *Session 6* • *‘The possibilities of taking control are quite limited when it comes to infections and deterioration of the lungs. Regarding this I had some issues with this text.’* • *‘At part 3, step 1, getting a healthier sleeping ritual; all electronics/screens off when it is time for bed. I think in general the advice is to turn these off 1 to 1.5* *h before going to bed, because otherwise people sleep worse when they were sitting at a computer or laptop right before going to bed (blue light).’* *Session 7* • *“I found this a ‘confrontational’ section. Intense anxieties are mentioned as examples and I hope you guide this part extra well. It could also exacerbate the anxiety.”* *Session 8* • *‘There is nice list given of e-health possibilities for follow-up treatment. I think that list also needs face-to-face treatment put in there as a possibility to discuss with the psychologist who guided this training.’*

### 3.3 Closed and open-ended feedback regarding specific features of eHealth CF-CBT

The video introduction created for the program was overall rated as clear and understandable (97%). The majority of participants thought the video was appealing (75%), however some participants commented that the colors and emotions of the characters could have been more subdued and gender neutral. Most thought the homework diary functioned well (78%), although some commented that access to the diary function could have been clearer. Participants understood the relaxation exercises (97%) and thought they were helpful (70%). All participants agreed the exercise on behavioral activation was well explained (100%) and the majority indicated it was useful (92%). Overall, participants understood the explanations regarding how to identify maladaptive thinking patterns (94%) and agreed that the exercises related to cognitive coping skills were useful (88%). Participants also stated that they found the perspectives of other patients with CF (i.e. direct quotations by adults with CF regarding their experiences specifically relevant to content and integrated into the CF-CBT program) to be very useful, increasing their sense of being motivated and providing support.

Although qualitative evaluation was generally positive (Table 3), participants provided feedback about specific pictures, text and functionality features that were not effective, which were subsequently changed. For example, a few patient participants pointed out that photographs depicting children may not be appropriate because many individuals with CF struggle with infertility or questions around their ability to start a family. In response to feedback, the developers removed pictures of children, provided a better balance of photographs of women and men, reduced the amount of text in several sections, and revised lay-outs to improve clarity. Improvements to functionality included creating additional diaries for homework assignments and ensuring patient access to program information after completion. The difficulty of fully evaluating the program by working through the web-based content without experiencing the “personal contact and guidance” inherent in both the synchronous and asynchronous therapist-guided elements of eHealth CF-CBT, was noted. In addition, the potential for participants to need “some attention for problems outside of CF” was raised.

### 3.4 Evaluation of individual eHealth CF-CBT sessions

Each of the 8 sessions was evaluated separately using the eHealth CF-CBT Feedback Questionnaire designed for this study; the pooled quantitative results of overlapping questionnaire items for each session are summarized in Table 4. When patient and provider participant ratings of all 8 sessions are combined, nearly all (98%) indicated that they “agreed” or “totally agreed” with the statement “I understand the session's subjects”. A high proportion of participants rated that they “agreed” or “strongly agreed” with the following statements: “The amount of text is good” (82.8%); “The use of language is good” (90.7%); “The perspectives of people with CF are useful” (80.7%); “The pictures are suitable” (73.8%); “Functions work well” (93.4%); and “Homework is clear” (96.4%), see Table 4.

**Table 4:** Evaluation of eHealth CF-CBT session elements.

Statement	Group	Strongly disagree	Disagree	Neutral	Agree	Strongly agree
		%	%	%	%	%
1. I understand the session's subjects	Total	0.4	0	1.2	46.5	52.0
	Patients	0.8	0	1.6	42.2	55.5
	Healthcare providers	0	0	0.8	50.8	48.4
2. The amount of text is good	Total	0.4	3.5	13.3	51.2	31.6
	Patients	0.8	3.9	17.2	43.0	35.2
	Healthcare providers	0	3.1	9.4	59.4	28.1
3. The use of language is good	Total	0.4	2.7	6.3	51.6	39.1
	Patients	0	0.8	8.6	43.8	46.9
	Healthcare providers	0.8	4.7	3.9	59.4	31.2
4. The perspectives of people with CF are useful	Total	0.5	3.7	15.1	38.0	42.7
	Patients	1.0	3.1	17.7	33.3	44.8
	Healthcare providers	0	4.2	12.5	42.7	40.6
5. The pictures are suitable	Total	0	5.9	20.3	54.7	19.1
	Patients	0	5.5	20.3	52.3	21.9
	Healthcare providers	0	6.3	20.3	57.0	16.4
6. Functions work well	Total	0	2.4	4.3	46.1	47.3
	Patients	0	3.9	1.6	35.9	58.6
	Healthcare providers	0	0.8	7.0	56.3	35.9
7. Homework is clear	Total	0.4	0.9	2.2	56.7	39.7
	Patients	0.9	0.9	2.7	51.8	43.8
	Healthcare providers	0	0.9	1.8	61.6	35.7

Note. Percentage of participants agreeing with statements regarding the 8 sessions. Reported percentages are calculated by averaging responses regarding each of the 8 sessions from questionnaires completed by each participant subgroup and the pooled total of 32 participants.

## 4 Discussion

The development of eHealth CF-CBT addresses an urgent need for new approaches to prevent and treat depression and anxiety in adults with CF. An early intervention approach to symptoms of depression and anxiety has the potential to reduce suffering while improving quality of life and adherence to CF care (Quittner et al., 2014; Smith et al., 2010; Verkleij et al., 2018).

The eHealth CF-CBT program is the first therapist-guided internet-delivered intervention for adults with CF. Results of this initial evaluation study suggest that the initial version of the eHealth CF-CBT program had adequate usability and functionality and was highly acceptable to both patients and healthcare providers. Feedback and suggestions to improve usability, functionality, presentation of information, and acceptability from this study directly informed program improvement. From its earliest stages, the program has been created including individuals with CF in a central role guiding project development and piloting, to ensure that the intervention is tailored to the diverse needs of adults with CF.

Given the burden of care and barriers to access to evidence-based mental health treatment for individuals with CF, an online treatment option will be particularly beneficial. An internet-delivered option may lessen the burden of having to travel long distances to the CF center for appointments; decrease wait times that would ordinarily exist for external mental health referrals and allow for continuity of mental health care during inpatient hospitalizations for CF exacerbations. Individuals with CF are closely followed by their CF Center and eHealth CF-CBT can be easily integrated into their CF care. People with CF may also more easily engage with mental health care that is provided by a member of their healthcare team who is familiar with the unique medical and psychosocial challenges they face. Individuals with CF and those that are post-transplant face a risk of cross-infection when they attend appointments in a healthcare setting. Due to COVID-19, in a period when anxiety and depression are possibly increased (Choi et al., 2020; Wei et al., 2020; Zhou et al., 2020), there is currently an especially high need for internet interventions from home, to reduce exposure to infection.

Nonetheless, there are also adults with CF for whom the eHealth CF-CBT program might not be appropriate. For example, those with severe symptoms of anxiety and depression or suicidality may need more intensive and entirely in-person treatment options. The eHealth CF-CBT program incorporates synchronous (in-person or telehealth) sessions as well as close monitoring and feedback of self-guided sessions to determine on an ongoing basis the appropriateness of the intervention or need for increased synchronous visits. More than 8 sessions of CF-specific CBT-based modules may also be needed to address symptoms of depression or anxiety or mental health comorbidities. The CF-CBT and eHealth CF-CBT programs may be useful as a first line intervention to engage people with CF with mild to moderate symptoms in care, acting as a bridge to follow-up therapy and/or psychiatric medication treatment for a subset.

The eHealth CF-CBT program was beta tested with people with CF and healthcare providers, but not specifically with adults with CF in the target range for depression and/or anxiety. Generalizability may be limited by the relatively small sample with mostly female participants; qualitative testing with additional male participants would be valuable. Further testing in an upcoming pilot study in the Netherlands supported by the Dutch CF Foundation, with adults with CF who endorse mild or moderate depression or anxiety symptoms following screening in the CF clinic, will be needed to confirm the usability and acceptability of the program, particularly with male participants. The pilot study of eHealth CF-CBT will explore the effectiveness of this internet-delivered blended-care program for improving depression, anxiety and quality of life, and inform the design of a larger randomized controlled trial.

The implementation of annual screening for depression and anxiety (Quittner et al., 2016) in US and European Centers provides a tremendous opportunity to intervene in response to early signs of distress and prevent negative psychological and health outcomes (Smith et al., 2016). A CFF/ECFS joint report on the current status of mental health care delivery in CF, however, concluded that additional training and educational resources are necessary to prepare CF centers for guideline implementation. Notably, there was substantial interest in receiving more mental health training, with 47% of providers indicating a desire for training in CBT (Abbott et al., 2015). Development of the CF-CBT and eHealth CF-CBT programs provide resources for CF centers to be able to provide CBT-based interventions to patients with CF. Dissemination of the eHealth CF-CBT program will require training providers in this model. The rapid, high uptake of telehealth necessitated by COVID suggests that this might be possible on a wide scale (Figueroa and Aguilera, 2020).

Lessons learned from the development of eHealth CF-CBT highlighted the value of creating interventions that are tailored to the needs of individuals with CF with input from the CF community. The process of program development and iteration with integration of feedback, although time and labor intensive, was critically important. The qualitative feedback of the participants was mainly positive, validating the goal of the developers of the program to normalize, destigmatize, and increase engagement in mental health care for people with CF. Session-specific feedback from key stakeholders led to strengthening the program by further decreasing the amount of text, eliminating jargon, increasing the amount of highly interactive and individualized exercises, highlighting key points visually, and including more of a balance of pictures of women and men to aim to improve identification and engagement of male participants. The process of reviewing the material and responding to participant input during self-management sessions will require a stepped care approach.

Overall, the eHealth CF-CBT blended care model for indicated preventative intervention and treatment has the potential to increase access to evidence-based mental health care for an at-risk group of individuals with chronic medical illness. The model can be integrated into the collaborative care models in use at CFF accredited centers in the US, and into existing European practices of stepped care for depression and anxiety. Taking into account differences in health care systems, such as insurance models, access to integrated psychological care, and referral pathways, will be important as dissemination progresses. We anticipate that integration of this intervention model into existing systems of care will positively impact adults with CF by providing evidence-based intervention for depression and anxiety, customized to each individual's specific needs, while minimizing cost and burden of care for patients and health care systems.

## Declaration of competing interest

The authors declare the following financial interests/personal relationships which may be considered as potential competing interests: This study was funded by Vertex Pharmaceuticals, Inc. via Circle of Care charitable awards and by research grants from the Cystic Fibrosis Foundation Therapeutics, Inc. and the Dutch Cystic Fibrosis Foundation. AMG reports grants and personal fees from Vertex Pharmaceuticals, outside the submitted work; grants, personal fees and non-financial support from the Cystic Fibrosis Foundation, outside the submitted work; personal fees and non-financial support from Cystic Fibrosis Australia, outside the submitted work; non-financial support from the European Cystic Fibrosis Society, outside the submitted work; personal fees from Johns Hopkins University/DKBmed, outside the submitted work.
